# Behavioral Disassociation of Perceived Sweet Taste Intensity and Hedonically Positive Palatability

**DOI:** 10.1523/ENEURO.0268-20.2020

**Published:** 2020-10-27

**Authors:** Esmeralda Fonseca, Vicente Sandoval-Herrera, Sidney A. Simon, Ranier Gutierrez

**Affiliations:** 1 Department of Pharmacology, CINVESTAV, Laboratory of Neurobiology of Appetite, Mexico City 07360, Mexico; 2 Departamento de Fisiología, Escuela Nacional de Ciencias Biológicas, Instituto Politécnico Nacional, Wilfrido Massieu, Mexico City 07738, México; 3 Department of Neurobiology, Duke University Medical Center, Durham, NC 27710

**Keywords:** intensity, palatability, sucrose, sweetness, taste quality

## Abstract

The intensity of sucrose (its perceived concentration) and its palatability (positive hedonic valence associated with ingestion) are two taste attributes that increase its attractiveness and overconsumption. Although both sensory attributes covary, in that increases in sucrose concentration leads to similar increases in its palatability, this covariation does not imply that they are part of the same process or whether they represent separate processes. Both these possibilities are considered in the literature. For this reason, we tested whether sucrose’s perceived intensity could be separated from its hedonically positive palatability. To address this issue, rats were trained in a sucrose intensity task to report the perceived intensity of a range of sucrose concentrations before and after its palatability was changed using a conditioned taste aversion (CTA) protocol. We found that the subjects’ performance remained essentially unchanged, although its palatability was changed from hedonically positive to negative. Overall, these data demonstrate that sucrose’s perceived intensity and its positive palatability can be dissociated, meaning that changes of one taste attribute render the other mostly unaffected. Thus, the intensity attribute is sufficient to inform the perceptual judgments of sucrose’s concentrations.

## Significance Statement

Subjects trained to classify sucrose concentrations could guide their decisions based on their perceived intensity and/or palatability. Despite the fact that both taste attributes covary with changes in sucrose concentration, we found that it was possible to change its hedonically positive palatability attribute without affecting the sensitivity to identify sucrose’s perceived intensity. That is, the subjects perceptual judgments of sucrose concentrations were based on the information provided by the intensity attribute showing, for the first time, that the hedonically positive aspect of sucrose’s palatability and its perceived intensity could be dissociated.

## Introduction

Mammals, including rodents and humans, have the impressive ability to identify and consume sweet-tasting, energy possessing, molecules, such as sucrose, and to avoid bitter-tasting molecules they find aversive. The consumption of sweet-tasting molecules to humans generates a taste percept that consists of a stable component, the tastant quality, which is presumed to remain constant (Staszko et al., 2020), and a variable component, its palatability (or hedonic value), whose value is dependent on a variety of factors including its internal states and learning experiences (Grill and Norgren, 1978a; Garcia et al., 1985). It is the palatability component that elicits the activation of oromotor responses that result in the consumption or rejection of foods (Grill and Norgren, 1978b; Berridge and Grill, 1983). One tried and tested way to change a tastant’s palatability is through a conditioned taste aversion (CTA), where a tastant is paired with an unpleasant agent (Grill and Norgren, 1978a). In virtually all of these CTA experiments, a single tastant concentration is tested (Domjan, 1975; Ninomiya et al., 1984; Kotlus and Blizard, 1998) and, although for sucrose, it is assumed the quality (identity) remains unchanged with changes in concentration (see above), the perceived intensity has not been dissociated.

In rodents, the palatability of sucrose has been measured using a brief access test by counting the number of sucrose-evoked licks in a small time window (Davis, 1973; Spector and Smith, 1984; Spector et al., 1998; Dotson and Spector, 2004), or using a taste reactivity test to record orofacial movements, such as gaping and tongue protrusions (Grill and Norgren, 1978b,c; St. John and Spector, 2008; Berridge et al., 2009). As noted, both oromotor palatability responses and the perceived intensity increase as the concentration of sucrose increases. However, the perceived intensity attribute cannot be directly measured by licking responses per se because it does not give information about the subject’s perception. For this reason, taste intensity discrimination tasks are better suited to obtain a judgment report of the perceived intensity of a tastant (Colbert et al., 2004; MacDonald et al., 2012; Fonseca et al., 2018). Here, we performed behavioral studies to determine whether the perceived intensity of sucrose is affected by giving a CTA that causes its palatability to be shifted from positive to negative.

In the rodent primary gustatory cortex (GC), electrophysiological recordings revealed competing viewpoints regarding how quality and palatability are encoded. Some studies, in which usually single concentrations of tastants were delivered to the animals, either passively (via an intraoral cannula) or actively (head-fixed licking), revealed that the processing of taste quality and palatability occurs serially with quality occurring first (Katz et al., 2001; Jezzini et al., 2013; Levitan et al., 2019). Using optogenetic manipulations, other studies found that tastant identity and palatability are processed by distinct channels or circuits (Wang et al., 2018). Specifically, Wang et al. (2018) were able to reverse the hedonic value of a sweet or bitter tastant by stimulating distinct insular cortex (IC) projections to the amygdala. They showed that mice with silenced neurons in the amygdala no longer exhibited oromotor palatability responses usually evoked by sweet-tasting or bitter-tasting chemicals, as perceived by humans. Interestingly, this occurs without affecting the ability of the same mice to recognize the quality of these tastants, thus suggesting that palatability and taste quality are two separate processes that can be dissociated (Wang et al., 2018). Of course, different anterior-posterior regions of the IC and their specific projections to the amygdala (and other regions) could also play a broader role in processing emotional states, such as pleasure and disgust-related facial expressions, beyond just conveying pure taste information (Dolensek et al., 2020). However, whichever the taste coding process proves to be correct, it is currently unknown whether palatability and the perceived intensity of sucrose solutions could be dissociated, and to what extent the removal of one orosensory attribute would impact the other.

Our goal was to determine whether the perceived intensity of sucrose could be behaviorally disentangled from its hedonically positive palatability attribute and, if it could, then determine its impact on the animal’s sensitivity to identify sucrose concentrations. This was accomplished using a novel behavioral approach that combined a sucrose concentration discrimination-generalization task to evaluate its perceived intensity and a CTA protocol to change its palatability from positive to negative. In sum, we found that the perceived intensity attribute was sufficient to inform the subjects about the sucrose concentration, indicating that the hedonic evaluation of a taste stimulus is dissociable from its intensity-discriminative properties.

## Materials and Methods

### Animals

We used nine male Sprague Dawley rats weighing 300–320 g. Animals were individually housed in standard laboratory cages in a temperature-controlled (22 ± 1°C) room with a 12/12 h light/dark cycle (lights were on 7 A.M. and off at 7 P.M.). All procedures were approved by the Institutional Animal Care and Use Committee. Rats were given *ad libitum* access to Chow food (PicoLab Rodent Diet 20) in their home cage. Water was available for 30 min after discrimination/generalization sessions, 45–60 min during CTA (see below for details), and 24 h during the two-bottle preference test. All experiments were performed from 1 to 4 P.M., since we found rats more alert and motivated to work during this period.

### Sapid stimuli

Sucrose was reagent-grade chemical quality purchased from Sigma-Aldrich (Mexico). It was dissolved in distilled water, and the following concentrations were used: 3, 4.75, 7.5, 11.75, and 18 wt/vol% (Fonseca et al., 2018). Solutions were freshly prepared every other day and maintained under refrigeration. Solutions were used at room temperature.

### Behavioral equipment

Animals were trained in four identical standard operant conditioning chambers of internal dimensions 30.5 × 24.1 × 21.0 cm (Med Associates Inc.; Fonseca et al., 2018). The front panel of each chamber was equipped with one central and two lateral V-shape licking ports with a photobeam sensor to register individual licks (Med Associates Inc.). Each port had a licking spout that consisted of either one (for lateral ports) or a bundle of up to six (for the central port) blunted needles (20-gauge) that were carefully sanded and glued at the tip of a stainless-steel sipper tube. Each needle was connected to a solenoid valve (Parker) via a silicon tube. The drop volume was calibrated before each session and maintained by using an individual and constant air pressure system (Fonseca et al., 2018). On the rear panel, there was an ambiance masking noise amplifier with a speaker that was turned on during the entire sessions to reduce external sounds from valves opening. A light was located on the front wall of the box. Chambers were enclosed in a ventilated, sound-attenuating cubicle. Experimental events were controlled and registered by a computer via a Med Associates interface (Med Associates Inc.).

#### Sucrose discrimination and generalization protocols

During discrimination sessions, the subjects were trained to produce differential responses based on the concentration of one drop of sucrose: to go to the left if low sucrose (3%) was received and go to the right if it was high (18%). These conditions were counterbalanced across subjects. The task comprised five epochs: return, pre-cue, cue-dependent licks (CDL), response, and outcome (also see Fonseca et al., 2018). The lights were on, and a trial began when an animal moved from the lateral port to the central port and emitted one dry lick (return). After two to three dry licks (pre-cue), the subject received a 10-μl drop of sucrose solution. Subsequent licks were dry and had no programmed consequences. The time spent licking in the central port from cue delivery onset comprised the CDLs epoch. Next, after sampling the cue, the subjects had to move from the central to a lateral port and emit one dry lick. If the animal chose the correct port (i.e., left → low, right → high), it received three 10-μl drops of water; otherwise, the lights went off for 50 ms. There was no fixed time limit to emit a response; although to initiate a new trial, a single lick in either port was required. Once the animals reached the learning criterion, defined as four consecutive sessions with ≥75% correct responses, generalization sessions were introduced. In these sessions, 80% of the trials were discrimination trials, which were identical to the trials during discrimination sessions; the remaining were generalization trials, in which one of five sucrose concentrations (3%, 4.75%, 7.5%, 11.75%, and 18%) or water (0%) were delivered, and the animals had to report whether they perceived it as low or high. The perceived intensity was defined as the percentage of responses emitted in the high-sucrose spout. In order to avoid any perceptual bias, no responses during generalization trials were rewarded (Fonseca et al., 2018). Once the response was emitted, the lights went off for 50 ms. Discrimination and generalization trials were pseudo-randomly interleaved, such that discrimination trials always flanked one generalization trial; the same was true for generalization sessions: they were preceded and followed by discrimination sessions. Flanking generalization trials and sessions, by discrimination trials and sessions, respectively, ensured a stable stimulus control intra and between sessions, as seen in the organized increase of high-sucrose responses as a function of sucrose concentration. Both discrimination and generalization trials lasted 20 min independently of the number of trials completed. The subjects had 30 min of access to water after discrimination and generalization sessions.

**Figure 2.**
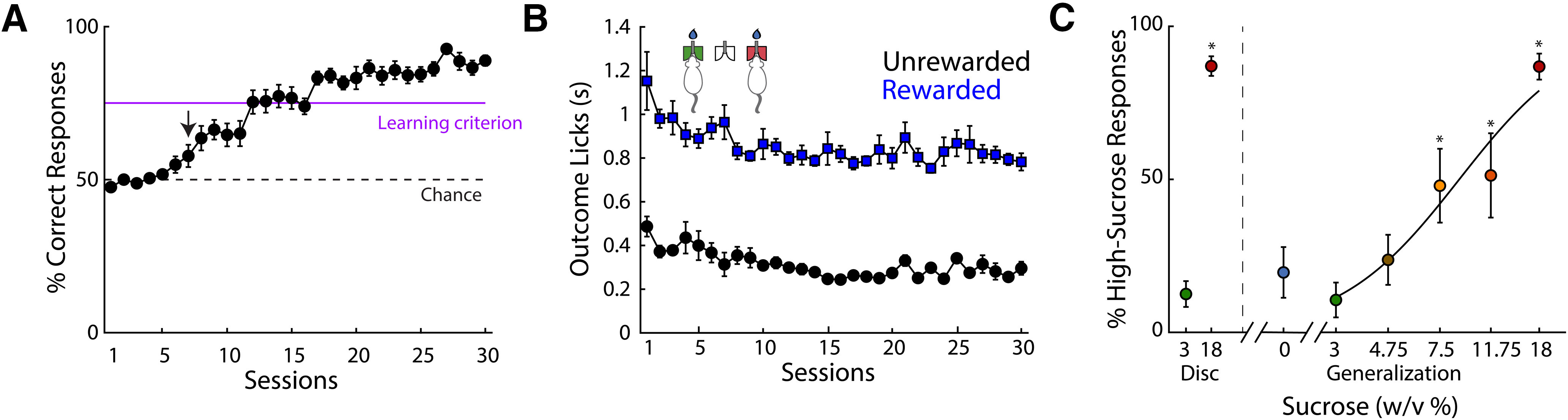
Subjects guide their decisions based on sucrose’s intensity. ***A***, Performance of subjects across discrimination (disk) sessions. After seven sessions, their performance in distinguishing 3% and 18% sucrose was above chance level (dashed line), while learning criterion (purple line; ≥75% correct responses during four consecutive sessions) was reached in 19 ± 4 sessions. The arrow at day 7 indicates that performance was significantly different compared with day 1. ***B***, Duration of lateral outcome epoch during rewarded (blue) and unrewarded (black) trials. From day 1, licking was significantly longer in rewarded than in unrewarded trials. ***C***, Percentage of responses where the subject perceived a sucrose concentration as high, during discrimination (disk) trials on the left side of the dashed line and generalization trials from the vertical gray dashed line to the right side. The *x*-axis is scaled logarithmically. * Significantly different from 3%-discrimination trials. Data are presented as mean ± SEM. For other behavioral measurements that were not significantly affected by the sucrose concentration, see Extended Data Figure 2-1.

#### CTA

Once the animals learned to guide their choices using the sucrose concentration as a cue, they underwent a CTA protocol to shift sucrose’s positive hedonic value into a negative one. Initially, over three consecutive days, the animals had 1-h access to water (baseline). The following day, they had access to one bottle filled with sucrose 18%, and after 15 min, they received an intraperitoneal injection 7.5 ml/kg of 0.2 m LiCl and then had access to water for 45 min (CTA-1). The animals were then allowed to recover for 1 d and had 1-h access to water. The following day, they went through a test 1 + CTA-2, where they were allowed to drink sucrose 18% for 15 min followed by a second intraperitoneal LiCl injection and then had 45-min access to water period. Animals were allowed to recover for the next 24 h with access to the water for 1 h. Finally, on the next day, the animals received 15 min of access to 18% sucrose, followed by 45 min of access to water (test 2). After they were given two CTAs, the animals were given 3 d of free access to water; afterward, their performance was re-evaluated in the sucrose intensity task following the same protocol described above.

#### Two-bottle preference test

A two-bottle preference test was performed to confirm that sucrose maintained a negative valence after re-evaluation in the sucrose intensity task. Briefly, the subjects had three baseline days with *ad libitum* access to water. In the next 2 d, they had 24 h of access to two bottles, one with sucrose 18% and the other with water, and each bottle was available on each side of their home cage. The bottle positions were counterbalanced across days.

### Data analysis

All data analysis was performed using MATLAB 2019b (The MathWorks Inc.) and GraphPad Prism 8. Data obtained from discrimination and generalization sessions are presented as mean ± SEM and were treated with parametric statistics. To compare the impact of the CTA in the discrimination and generalization measurements, the data from the five discrimination/generalization sessions before CTA were concatenated and compared with the concatenated data from the five sessions after CTA. Differences among the data mentioned above were identified using parametric statistics. Data obtained during the CTA protocol and the two-bottle test are presented as median ± interquartile range (iqr) and were treated with non-parametric statistics, since only one observation per subject was obtained, and the size of the groups was relatively small (*n*_saline_ = 4, *n*_LiCl_ = 5). Unless otherwise indicated, α level was set up at 0.05. All statistical results can be found in the Extended Data Table 1-1.

10.1523/ENEURO.0268-20.2020.t1-1 Extended Data Table 1-1 Statistical table. Download Table 1-1, XLSX file.

#### Sucrose intensity task: discrimination and generalization

To identify whether there was a significant difference in performance across sessions, a mixed-effects model was employed (Bagiella et al., 2000). To identify when the performance started to be significantly different from the first day, we used Dunnett’s multiple comparisons test (Dunnett, 1955). Changes in CDL, response, return, outcome differences by session, concentration (low vs high), or interaction (session × concentration) were assessed by a mixed-effects model (Bagiella et al., 2000). The same approach was applied to licking given in the outcome epoch divided by rewarded versus unrewarded trials instead of concentration. The generalization curves were obtained by quantifying the number of responses emitted to the port associated with high sucrose for each sucrose concentrations given during the generalization trials and fitting the following four-parameter sigmoidal function to those data points:

$$\text{P}\left(\text{High}\right)\;=\;y_{0}\;+\;\frac{a}{\text{1}\;+\;{\text{e}}^{\left(\left(xo-x\right)\;*b\right)}}$$

where *y_o_* is the left endpoint, *a* is the asymptote (minimum and maximum high-sucrose responses), *x_o_* represents the point of the inflection of the curve, *x* is the log_10_ sucrose concentrations, and *b* is the slope. The psychophysical measurements were obtained from the fitted curve: point of subjective equality (PSE) is the concentration that elicits 50% of high-sucrose responses, that is, the concentration that is equally likely to be perceived as “low” or “high.” Limen (Li) is defined as the difference between the concentrations that elicited 75% and 25% of high-sucrose responses divided by 2, indicates the amount of concentration that needs to be changed to perceive that a change in the stimulus has occurred. Finally, the Weber fraction (WF), which is a sensitivity measure, was computed as from the division of the Li over the PSE. In contrast to the just noticeable differences (JNDs) protocol, where only one standard concentration is used (Colbert et al., 2004), in our task, when the rats are presented with the test-stimuli (a drop of sucrose), they then need to report the similarity of the test stimulus to either the lower (3%) or the higher (18%) standard concentration tested. This approach is similar to what has been employed in temporal bisection tasks (Church and Deluty, 1977).

Differences in the sucrose-high classification responses from the generalization sessions were analyzed using a one-way ANOVA, followed by Dunnett’s multiple comparisons test using responses during the 3% discrimination trials as control.

#### CTA

To confirm significant differences in the amount of liquid consumed during the first 15 min of liquid access, a Mann–Whitney’s *U* test was performed (Kerby, 2014).

#### Sucrose intensity task re-evaluation

To identify differences in the performance, CDL, response time, return time, and the number of trials because of sucrose devaluation, the value of these behavioral measurements during the five pre-CTA versus the five post-CTA discrimination sessions were compared using a Student’s *t* test. The same approach was used to detect significant changes in the PSE, Li, and WF during the five generalization sessions pre-CTA versus post-CTA. Sucrose-high classification responses in each group (saline or LiCl) was compared before and after the treatment by a two-way ANOVA.

#### Two-bottle preference test

The preference ratio (PR) was defined as the sucrose intake divided by the total (water + sucrose) intake during a 24-h period. The differences in the PR between saline and LiCl groups were tested using Mann–Whitney’s *U* test.

## Results

**Figure 1.**
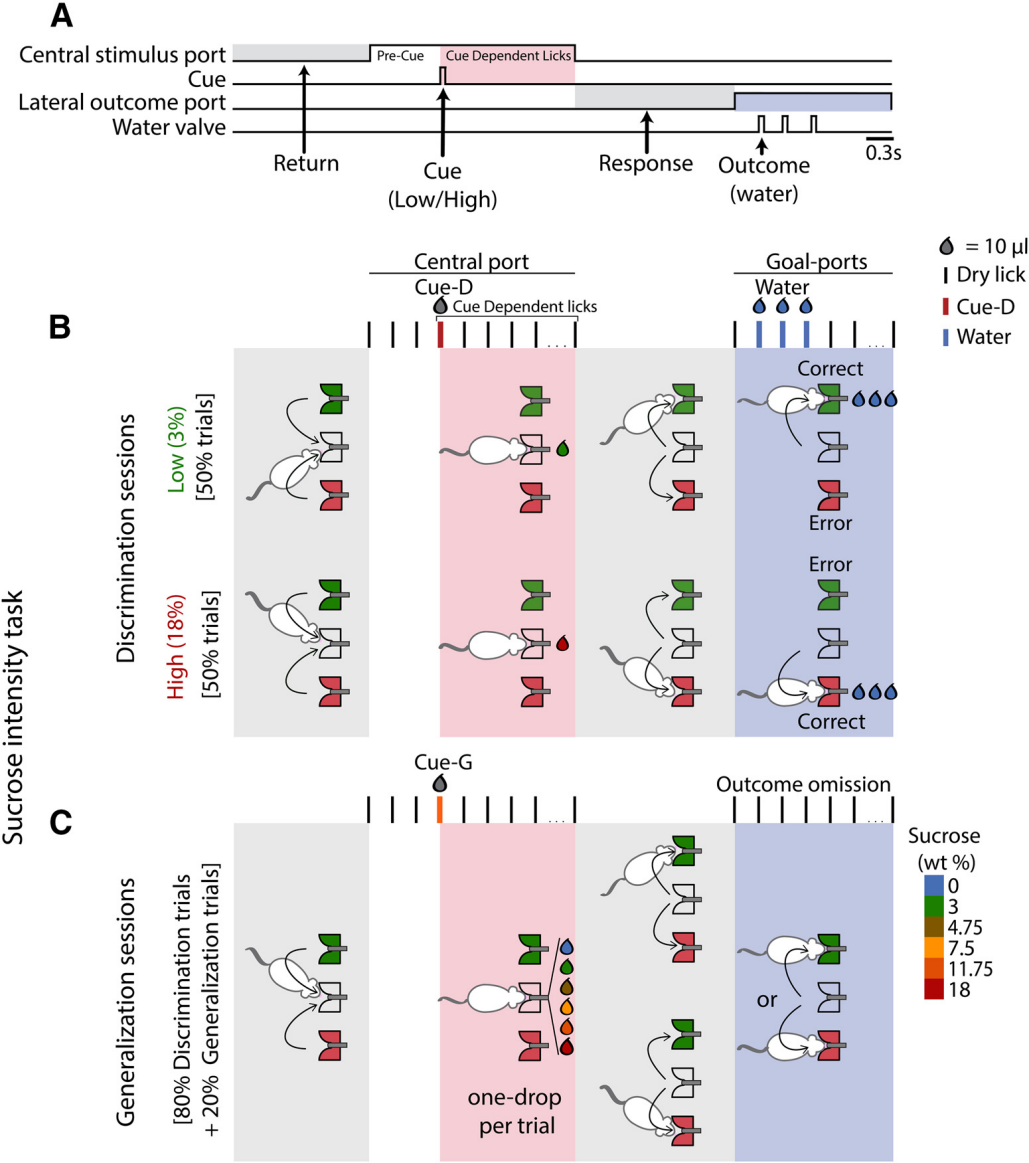
Structure of the sucrose intensity task. ***A***, A schematic of the task that is comprised of discrimination (***B***) and generalization (***C***) sessions. ***B***, During discrimination trials, the subjects moved toward the central port (return epoch) to lick and receive a single 10-μl drop of either a 3% sucrose-low (green) or 18% sucrose-high (red). The licks emitted in the central port comprise the cue epoch. The animals had to emit three dry licks before receiving the cue (pre-cue) and then produce CDL before detecting whether the concentration was high or low by going to the left side for a low concentration or to the right side for a high concentration (response epoch), counterbalanced across subjects. For making a correct response, and after one dry lick in the chosen lateral port, they would receive three drops of water. For incorrect choices, the trial was ended (outcome epoch). ***C***, Generalization sessions were composed of 80% discrimination trials (cue-D) and 20% generalization trials (cue-G). The trial structure of generalization trials is the same as in discrimination, but with two main differences: (1) the subjects received one drop of either six sucrose concentrations (0%, 3%, 4.75%, 7.5%, 11.75%, 18%); and (2) no reward was given during the outcome epoch.

In the sucrose intensity task, rats were trained to lick a central spout and then report whether they perceived 3% versus 18% sucrose concentration either as “low” or “high” by responding on the left or right port, respectively (Fig. 1*A,B*). Briefly, the task was divided into five epochs: return, where the animals return from lateral to the central port to initiate a trial; pre-cue (dry licks gave before cue delivery); CDL, where the animals emitted the dry licks evoked by cue delivery; response, where the animals moved from the central to a lateral port to collect the reward; and outcome, where animals were given a correct response, licked a lateral spout to obtained three drops of water as a reward. After learning this intensity task, we introduced generalization sessions, where the animals were now required to generalize their responses to multiple sucrose concentrations between 3% and 18% sucrose (Fig. 1*C*).

### Acquisition of the sucrose intensity task

In the intensity task the animal’s performance increased until they achieved ≥75% correct responses after 19 ± 4 sessions (Fig. 2*A*, the learning criterion, purple line). Dunnett’s multiple comparison test showed that compared with day 1, significant differences were achieved after day 7 (*p *=* *0.01; Fig. 2*A*, arrow). The CDLs time in seconds, which is a measure of sucrose’s palatability (Perez et al., 2013), decreased across sessions (*F*_(40,606)_ = 6.8, *p *<* *0.0001; Extended Data Fig. 2-1*A*). However, there were no significant effects of the concentration factor (*F*_(1,16)_ = 0.02, *p* > 0.05) or interaction sessions × concentration (*F*_(40,606)_ = 0.6, *p* > 0.05). The fact that CDLs were not modulated by sucrose concentrations could reflect a masking effect on palatability responses since sucrose also serves as a discriminative cue that directs operant responding motivated by water deprivation and water reinforcement. Likewise, the response movement (sessions *F*_(40,606)_ = 8.2, *p *<* *0.0001; Extended Data Fig. 2-1*B*) and the return movement (sessions *F*_(40,606)_ = 10.8, *p *<* *0.0001; Extended Data Fig. 2-1*C*) movement times significantly decreased early in training (from session 2 to 6). However, late in training, they increased and remained at the same level across sessions. Neither the concentration factor (response: *F*_(1,16)_ = 0.03, *p *>* *0.05; return: *F*_(1,16)_ = 0.7, *p *>* *0.05), nor a significant effect for interaction between factors were significant (response: *F*_(40,606)_ = 0.5, *p *>* *0.05; return: *F*_(40,606)_ = 0.5, *p *>* *0.05). In sum, in this sucrose intensity task, neither CDLs, response, or the return movement was dependent on the sucrose concentration.

10.1523/ENEURO.0268-20.2020.f2-1 Extended Data Figure 2-1 During discrimination sessions of the sucrose intensity task, other behavioral measurements were not significantly affected by the sucrose concentration. ***A***, Time spent licking in the central port after cue delivery (CDL). Note that CDL decreased over sessions to the same level for sucrose concentrations: low (green) and high (red). ***B***, Time spent to move from the central to the lateral port (response movement) across sessions. Same conventions as in ***A***. ***C***, Time needed to move from the lateral to the central port (return movement). ***D***, Time licking in the lateral port is similar in both sucrose concentration trials. Data are presented as mean ± SEM. Download Figure 2-1, TIF file.

With regard to the licks in the outcome epoch, there was a significant difference between rewarded and unrewarded trials (factor trial type; *F*_(1,16)_ = 394.9, *p *<* *0.0001; Fig. 2*B*), but no differences among concentrations were found (*F*_(1,16)_ = 0.02, *p* > 0.05; Extended Data Fig. 2-1*D*). Thus, subjects were highly sensitive to the outcome of the task. Rats detected the absence of reward in <300 ms (Fig. 2*B*, black circles; Fonseca et al., 2018). Furthermore, licking during both rewarded and unrewarded trials significantly decreased as a function of sessions (*F*_(40,606)_ = 3.85, *p *< 0.0001). Thus, rats reduce the time spent doing unnecessary licking, which they could use to increase the number of trials, thereby increasing the amount of water reward obtained per session.

### Generalization trials

Once the animals learned to discriminate a low (3%) from a high (18%) sucrose concentration, the generalization trials began with the discrimination trials interleaved with them (Fig. 1*C*). During generalization trials, the subjects were presented with water (0% sucrose) or one of five sucrose concentrations (3%, 4.75%, 7.5%, 11.75%, and 18%), which they had to classify as either “low” or “high.” Figure 2*C* shows a plot of the perceived sucrose concentration as “high” as a function of sucrose concentration (*F*_(7,64)_ = 78.2; *p *<* *0.0001). In comparison to the 3% sucrose-discrimination trials (*p *<* *0.0001; Fig. 2*C*, left side), subjects made significantly more “high” judgments of sucrose concentrations ≥7.5%. In sum, as reported by Fonseca et al. (2018), the subjects could make perceptual decisions about sucrose’s intensity. That said, we are aware of the possibility that animals could guide their decisions based on either sucrose’s intensity or its palatability since for sucrose both attributes covary (St. John and Spector, 2008). To distinguish between these possibilities, using a CTA, we altered the palatability of sucrose from appetitive to aversive.

### A CTA to 18% sucrose changes its palatability from positive to negative

**Figure 3.**
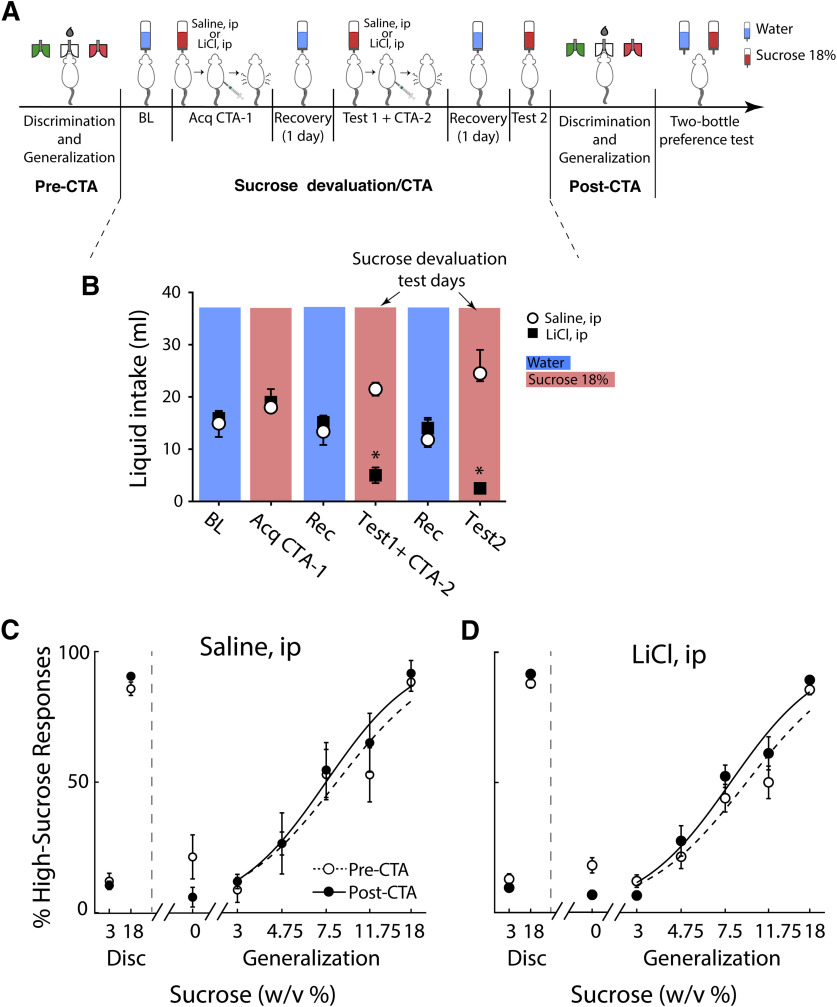
Devaluating sucrose’s hedonically positive palatability does not affect sucrose’s response to concentration (intensity). ***A***, Timeline of the experimental design. After the animals were trained to identify sucrose concentrations, as shown in Figures 1, 2 (discrimination disk and generalization; pre-CTA), the subjects went through a sucrose devaluation procedure involving two consecutive CTAs. As a control, a baseline water consumption (blue bottle) was measured over 15 min. The next day after baseline testing for water, the animals were given 18% sucrose (red bottle) to consume, and 15 min later, they received an intraperitoneal injection of 0.2 m lithium chloride (LiCl), a visceral malaise agent [Acquisition of first CTA (Acq CTA-1)]. Then, after a recovery day, the subjects were again given 18% sucrose for 15 min to consume followed by the second injection of 0.2 m LiCl or saline (test 1 + CTA-2). After a recovery day, test 2 was performed by measuring the intake of 18% sucrose. After the CTA protocol, sucrose intensity task was re-evaluated (pre-CTA vs post-CTA, see ***C***, ***D***). Finally, a sucrose preference test was conducted using a two-bottle test to confirm that it remained aversive at the end of the experiments (for results, see Fig. 4). ***B***, A histogram showing liquid intake for several of the epochs shown in dashed line expansion of ***A***; 15 min after liquid intake, rats were injected with saline (open circle) or 0.2 m LiCl (black rectangles). Blue and red background shadows depict conditions where subjects had access to water and sucrose, respectively. The two upper arrows indicate the two test days where it can be seen that subjects developed CTA. * Significant differences between saline and LiCl groups. Data are shown as median ± iqr. ***C***, Percentage of times the subject in the saline group classified a sucrose concentration as “high.” Performances obtained before and after CTA are represented by the open, and black filled circles, respectively. The dashed (pre-CTA) and solid (post-CTA) lines are the fitted sigmoid, respectively. The *x*-axis is scaled logarithmically. Data are presented as mean ± SEM. ***D***, Same as in ***C*** but for rats injected with LiCl. These results suggest that sucrose’s intensity attribute is the main orosensory feature used by rats to solve the task. Extended Data Figure 3-1 shows values for the other behavioral measurements, and Extended Data Figure 3-2 shows the performance of each individual rat.

In order to dissociate sucrose’s perceived intensity attribute from its palatability, we changed the palatability component. This was achieved by inducing a CTA using 7.5 ml/kg of 0.2 m LiCl (a visceral malaise agent) that was paired to the consumption of 18% sucrose. Figure 3*A* depicts the experimental protocol. Briefly, after training the animals in the sucrose intensity task (Fig. 2), they then received a CTA to 18% sucrose (Fig. 3*B*). This was then followed by re-evaluation of the intensity task (Fig. 3*C*). Finally, a two-bottle preference test between water and sucrose 18% was given (see below). Because of the extremely rewarding effects of sucrose (la Fleur et al., 2007; Lenoir et al., 2007), we repeated the CTA procedure twice to achieve a longer-lasting aversion (Fig. 3*B*). Furthermore, given that the CTA is stronger for the specific concentration used as a conditioned stimulus (Richardson et al., 1984), we paired LiCl against 18% sucrose, which is the most salient stimulus in the task. We found that the amount of water ingested during the first 15 min in the Baseline (BL) epoch, as well as the intake of sucrose before the acquisition of CTA, was the same in rats injected intraperitoneally either with saline or LiCl (BL: *U* = 8, *p *>* *0.05; sucrose: *U* = 7, *p *<* *0.05). However, once a CTA was established, the animals injected with LiCl consumed a significantly lower volume of 18% sucrose (5.0 ± 2.5 ml) than the saline group (21.5 ± 2.0 ml; *U* = 0; *p *<* *0.02; see test 1 + CTA-2 d). These differences were slightly larger on the second test day (test 2; *U* = 0, *p *<* *0.01). In sum, as expected, given a CTA successfully changed the hedonic value of 18% sucrose from appetitive to aversive.

### Changes in sucrose’s palatability left intensity-guided perceptual judgments practically unaffected

To determine whether the subjects could classify sucrose concentrations by their intensity after a CTA, we re-evaluated their sucrose intensity task performance. Although we observed a significant decrease in the percent correct performance before and after the change in palatability from appetitive to aversive for the CTA group (from 92.1 ± 0.8% to 89.2 ± 0.9% correct from pre-CTA to post-CTA, *t*_(23)_ = 2.72; *p *<* *0.0001; Table 1), all subjects continued performing well above the learning criterion after CTA (Extended Data Fig. 3-1). Moreover, the performance was not significantly different between saline post-CTA and LiCl post-CTA (*t*_(41)_ = 1.23; *p *>* *0.05), suggesting that changes in positive palatability did not affect the perceived intensity. Likewise, the performance pre-CTA versus post-CTA of the Saline group was unaffected (*t*_(16)_ = 0.89, *p* > 0.05). Extended Data Figure 3-1 and Table 1 show a detailed description of other behavioral measurements in the discrimination sessions unaffected by the CTA procedure. Concerning this study, we found that the percentage of animals judging sucrose as “high” as a function of sucrose concentration was unchanged from before to after CTA, for the saline (*F*_(1,30)_ = 0.16, *p > *0.05; Fig. 3*C*) and the LiCl (*F*_(1,40)_ = 0.65, *p* > 0.05; Fig. 3*D*) groups. Details of the PSE (that is, the concentration that it is equally likely to be perceived as “low” or “high”), Li (refers to the amount of concentration that needs to be changed to perceive that a change in the stimulus has occurred), and WF (a sensitivity measure) are found in Table 1. Briefly, only PSE decreased significantly for the saline (*t*_(19)_ = 2.85, *p *<* *0.05) but not for the LiCl (*t*_(17)_ = 0.68, *p *<* *0.05) group. On the other hand, the Li (saline *t*_(15)_ = 1.42, *p *>* *0.05; saline *t*_(15)_ = 0.85, *p *>* *0.05) and the WF (saline *t*_(15)_ = 0.91, *p *>* *0.05; saline *t*_(15)_ = 0.47, *p *>* *0.05) were not significantly affected by sucrose devaluation (Table 1; Extended Data Fig. 3-2). That is, the sensitivity to identify sucrose concentrations was unaffected by the CTA procedure. Therefore, to solve the task, subjects used the taste information contained in the perceived intensity attribute, which was essentially unaffected by changes in the hedonically positive component of palatability.

**Table 1:** Behavioral changes in sucrose intensity task after CTA to 18 w/v % sucrose

Behavioral measurements	Saline, i.p.		LiCl, i.p.	
	Pre-CTA	Post-CTA	Pre-CTA	Post-CTA
Performance (%)	87.7 ± 3	87.3 ± 2.7	92.1 ± 0.8	89.2 ± 0.9*
CDL (s)	0.5 ± 0.1	0.6 ± 0.1	0.6 ± 0.04	0.5 ± 0.04
Response (s)	0.8 ± 0.04	0.9 ± 0.04	0.8 ± 0.04	0.8 ± 0.05
Return (s)	1.8 ± 0.05	1.8 ± 0.02	1.9 ± 0.06	1.9 ± 0.05
Number of trials	249 ± 30.2	258.5 ± 13.7	270.2 ± 2.8	258.7 ± 8.6
PSE	9.6 ± 0.6	7.6 ± 0.4*	9.4 ± 3.3	8.6 ± 2.9
Li	4.7 ± 0.4	3.5 ± 0.4	3.3 ± 0.5	2.8 ± 0.4
WF	0.50 ± 0.05	0.42 ± 0.04	0.36 ± 0.05	0.33 ± 0.05

Data presented as mean ± SEM. Data averaged from the five last and first discrimination (from performance to number of trials) and generalization (PSE, Li, WF) sessions, before and after CTA procedure. * Indicates significant difference. All statistical results can be found in the Extended Data Table 1-1.

10.1523/ENEURO.0268-20.2020.f3-1 Extended Data Figure 3-1 Performance and other behavioral measurements were essentially unchanged after animals were given a CTA to 18% sucrose. ***A***, Correct responses of the last and first five discrimination sessions, before (open circles) and after (filled circles) CTA, for rats, injected intraperitoneally with saline (left) and LiCl (right). Each circle represents one session of each subject. The learning criterion is depicted by the purple line. ***B***, Time spent licking during the cue epoch (CDL). Same conventions as in ***A***. ***C***, Same as in ***B*** for the response movement time. ***D***, Same as in ***B*** for the return movement time. ***E***, Number of trials completed. Conventions are the same as in ***B***. Data are presented as mean ± sem. * Denotes statistical difference *p *<* *0.05. Download Figure 3-1, TIF file.

10.1523/ENEURO.0268-20.2020.f3-2 Extended Data Figure 3-2 ***A***, Percentage of times five subjects in the saline group classified a sucrose concentration as “high.” Averaged performance obtained during the five sessions before and after CTA is represented by the open, and gray filled circles. The dashed and solid lines are the fitted sigmoid, respectively (see Materials and Methods). ***B***, Same as in ***C*** but for rats injected with LiCl (black). The *x*-axis is scaled logarithmically. Data are presented as mean ± SEM. Download Figure 3-2, TIF file.

### The shift of 18% sucrose’s valence from positive to negative persists after re-evaluation on the sucrose intensity task

**Figure 4.**
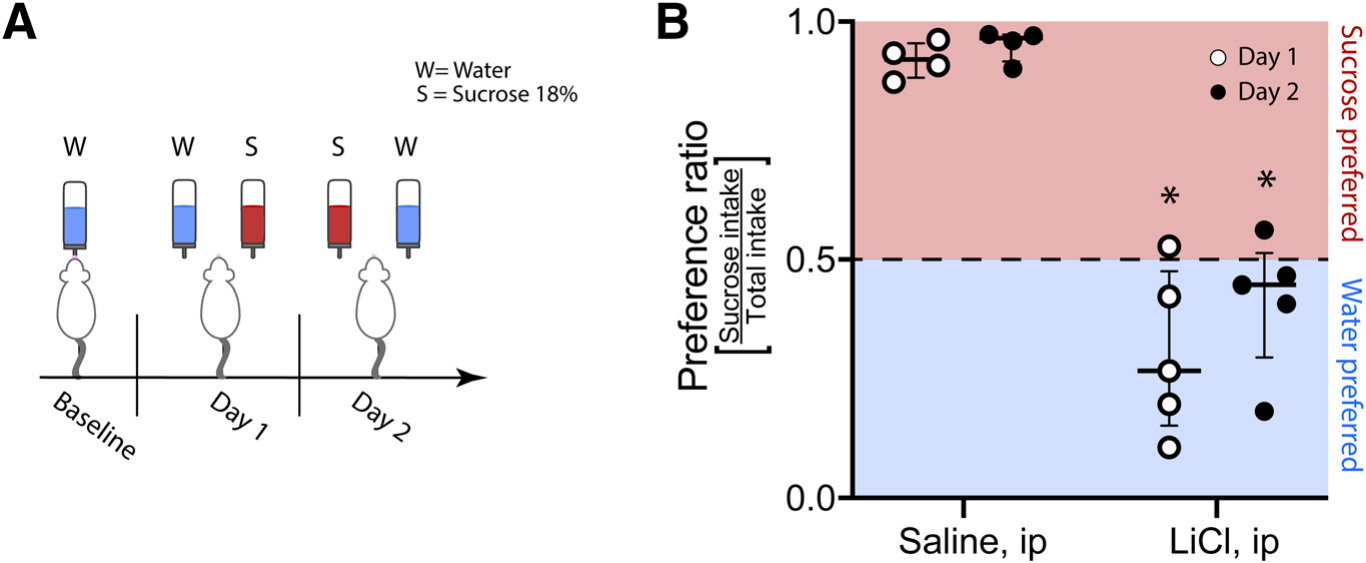
The CTA to 18% sucrose was not extinguished after re-evaluating performance in the sucrose intensity task (post-CTA; Fig. 3*A*). ***A***, Two bottle experiment design. ***B***, Sucrose PR for saline and LiCl groups during day 1 (white circles) and day 2 (black circles) of the two-bottle preference test. Note that, compared with saline treated rats, subjects injected with LiCl persistently disliked and rejected sucrose. * Significant differences compared with saline-treated rats. Data are presented as median ± iqr.

To demonstrate that in the sucrose intensity task, the rejection of sucrose remained until the end of re-evaluation, we performed a two-bottle preference test (water vs 18% sucrose; Fig. 4*A*). A PR was calculated by dividing the sucrose intake amount by the total intake of sucrose + water. Note that PRs from 0 to 0.5 indicate water was preferred, and PRs from 0.5 to 1 means 18% sucrose was preferred. For the saline and LiCl groups, the difference in PR during both testing days was significantly different (day 1: *U* = 0, *p < *0.02; day 2: *U* = 0, *p < *0.02; Fig. 4*B*). The control saline group displayed a high-sucrose preference that was consistent across days (day 1: 0.92 ± 0.06; day 2: 0.97 ± 0.04; *W* = 10, *p *>* *0.05). No subject showed sucrose preference for the LiCl group compared with the control group (Fig. 4*B*). The consumption pattern was similar during both test days (day 1: 0.27 ± 0.45; day 2: 0.27 ± 0.14; *W* = 9, *p *>* *0.05). Thus, 18% of sucrose remained aversive even after the subjects correctly classified sucrose concentrations during the re-evaluation test. That said, in this task, animals were using information about the concentration (intensity) rather than the hedonic palatability to make their taste perceptual decisions about sucrose’s perceived intensity.

## Discussion

The encoding of tastants consists of at least two processes that involve its quality and its palatability. The quality of sucrose is presumed to remain constant for all concentrations (Pfaffmann, 1941; Berridge and Grill, 1983; Richardson et al., 1984; Gutierrez et al., 2020; Staszko et al., 2020), whereas its palatability can change depending on a variety of factors including learning and the animal’s internal states (Rolls, 2007; Levitan et al., 2019). In the primary GC, there is a disagreement about whether taste quality is encoded by narrowly-tuned and spatially confined neurons (Chen et al., 2011) or by both narrowly-tuned and broadly-tuned neurons distributed throughout the GC and other cortical regions (Jezzini et al., 2013; Fletcher et al., 2017; Levitan et al., 2019). Regarding palatability, there is also disagreement as to its encoding in the GC. One group of studies indicated that palatability occurs in a serial fashion after the quality and that it can even use the same neurons to encode both features (Katz et al., 2001; Jezzini et al., 2013; Vincis et al., 2020). In contrast, another study found that quality and palatability are processed by independent circuits, and that palatability can be changed without changing taste quality (Wang et al., 2018). There is, however, a third sensory attribute also encoded in the GC, namely, the perceived intensity of a tastant. It is presumed that as the concentration is increased (decreased), its perceived intensity will follow (Gautam et al., 2012; MacDonald et al., 2012; Blonde et al., 2015), assuming that the quality remains unchanged as is the case for sucrose. In this study, we developed a protocol in which animals tasting solutions of sucrose reported their perceived intensity. Then, using a CTA to change sucrose’s palatability from hedonically positive to negative, we found that the perceived intensity remained unchanged, showing that these responses were primarily guided by its intensity and not by its palatability attribute. This indicates that the hedonic aspects of palatability, which are associated with the oromotor aspects of eating (Berridge and Grill, 1983), are not necessary for the animals to perceive the intensity of a tastant.

### Taste quality and palatability

Stimulating the tongue with sugars activates “sweet” taste receptor cells (TRCs and their associated brain circuits; Huang et al., 2006) that are then translated into multiple attribute representations informing the rodents about what we assume is similar to what humans experience as sweetness (Breslin, 2013; Wang et al., 2018; Gutierrez et al., 2020). Beyond expectation, this process is initiated with the presence of a chemical stimulus in the mouth and then with the formation of its quality and palatability attributes. Data in rodents currently indicate that these attributes could be formed either serially and/or in parallel. The serial model is supported by electrophysiological recordings (Katz et al., 2001; Levitan et al., 2019; Vincis et al., 2020) and suggests that these attributes are formed temporally with quality giving rise to palatability. Alternatively, one could also argue that a dynamic network of parallel circuits could provide information to GC differently over time. In this regard, the study by Samuelsen et al. (2013) found that pharmacological inactivation of ventroposteromedial nucleus of the thalamus (VPMpc; i.e., the primary gustatory thalamus) disrupted taste coding in a subpopulation of GC neurons although it was not completely eliminated. These findings support a parallel and distributed processing mechanism for inputs converging onto the GC. Moreover, the parallel processing model is also supported by the work on decerebrate rats (Grill and Norgren, 1978a). These animals were decerebrated at the level of the superior colliculus and thus do not have intact connections to the VPMpc that, in turn, projects to the GC, an area that plays a key role in gustatory processing, taste-guided decisions, taste-aversion learning, taste sensitivity and taste recognition (Yamamoto et al., 1980; Cechetto and Saper, 1987; Cubero et al., 1999; Kobayashi, 2011; Samuelsen et al., 2013; Blonde et al., 2015; Peng et al., 2015; Fletcher et al., 2017; Fonseca et al., 2018). Nevertheless, these animals still give normal hedonically positive oral responses to sucrose (Grill and Norgren, 1978a), indicating that palatability alone can drive basic oromotor reflexes elicited by sucrose. Although the forebrain is required to update new associative changes in palatability, it is important to note that in decerebrate rats, a CTA cannot be conditioned (Grill and Norgren, 1978a). Other evidence supporting, but not proving, that taste quality and palatability could be processed by different brain circuits comes from studies using TRPM5 knock-out mice. These mice are partially “blind” to compounds that humans perceive as sweet (Banik et al., 2018). They have a diminished ability to detect sucrose but could still learn a reduced, but selective, CTA to sucrose (Eddy et al., 2012). Interestingly, they showed a normal ability to develop a conditioned flavor aversion to a sweet and odor mixture, suggesting that these mice maintained the ability to update the palatability despite the impairment in taste detection (Eddy et al., 2012). Although these results have been interpreted as evidence for TRPM5-independent pathway to detect sweet tastants (Eddy et al., 2012; see also Banik et al., 2018), it could be argued that it also supports the existence of independent-parallel processing of palatability and taste quality. In this parallel model, after sugar activates the sweet responsive TRCs, the identity (quality) and the palatability should arise more or less in parallel, and no assumption is made that one attribute drives the other. Whichever hypothesis proves to be correct, it is clear that once taste attributes are formed, they then became independent because, as previously noted, sweet taste quality recognition can be dissociated from its palatability (Wang et al., 2018). Here, we extend these observations by demonstrating that sucrose’s perceived intensity and hedonically positive palatability can also be dissociated at the behavioral level.

### Dissociation of taste intensity from palatability

A third sensory attribute conveying information about a tastant is its intensity (Lawless and Skinner, 1979). In the periphery, increasing the concentration should activate more receptors and thus increase the intensity (Beidler, 1954; Wu et al., 2015). However, from a behavioral viewpoint, although related (Lawless and Skinner, 1979), the perceived intensity is not reflected by any licking responses per se, but rather it is a process that requires the animal to make a decision regarding whether one concentration of a tastant is higher or lower than a standard concentration. Using a sucrose intensity task combined with CTA, we found behavioral evidence supporting that the perceived taste intensity could be separately processed from the hedonically positive part of sucrose’s palatability (Fig. 3).

Although the reason(s) the gustatory system seems to process palatability distinctly from intensity is not known, one possibility is that it could result in a sharpening of aversions to a specific tastant and its concentration, rather than generalizing them to an entire class of foods with similar features. In this regard, intrachemical and interchemical CTA generalization studies had found that for a particular tastant, rats develop the strongest aversion to a given tastant concentration when it is paired with visceral malaise and exhibit smaller aversions to either lower or higher concentrations of the same tastant (Tapper and Halpern, 1968; Rozin et al., 1979; Richardson et al., 1984; Eddy et al., 2012). We argue that the same principle (i.e., the concentration of sucrose can be a cue) is used by rats to solve our sucrose intensity task because they can use 10-μl drops of 18% sucrose (maximum tested concentration) as a cue to classify it as high and to obtain a water reward, regardless of whether it was paired with malaise. Alternatively, and given that it is expected that the CTA to 18% sucrose would have a smaller effect on changing the palatability of lower 3% sucrose concentration (Richardson et al., 1984). In this regard, one possibility is that after CTA the rats guided their choices based solely on the intensity of the lower 3% concentration. However, we found this possibility to be improbable since they judged the intensity of 3% sucrose as “low” and the 18% sucrose as “high” in a comparable manner before and after CTA (Fig. 3*C*). The well-organized increase in high responses as a function of sucrose concentration suggests that concentration exerted and maintained stimulus control over the behavior before and after CTA. Thus, we conclude that the most parsimonious explanation of our results is that the hedonically positive palatability of sucrose is not needed to identify its intensity.

### Dissociation of taste quality from the intensity

To our knowledge, no attempt has been made to dissociate taste quality from the perceived intensity, although Gautam et al. (2012) found that intensity is the most crucial dimension to explain taste quality. However, in humans, neither taste adaptation nor cross-adaptation procedures produced changes in tastant quality (Meiselman, 1968). For example, after overstimulation with a high-sucrose concentration, it is possible to experience as less intense a lower sucrose concentration without affecting its sweet quality (Meiselman, 1968). This suggests that quality and intensity could also be separately processed, as suggested by our behavioral experiments for palatability. That is, the hedonically positive palatability responses evoked by sucrose are not necessary for animals to detect its perceived intensity.

## Synthesis

Reviewing Editor: Julie Bakker, University of Liege Decisions are customarily a result of the Reviewing Editor and the peer reviewers coming together and discussing their recommendations until a consensus is reached. When revisions are invited, a fact-based synthesis statement explaining their decision and outlining what is needed to prepare a revision will be listed below. The following reviewer(s) agreed to reveal their identity: Chad Samuelsen.

Although both reviewers agree that the results are certainly interesting and novel, some serious re-writing will be necessary for the ms to become acceptable for publication. Please find below the 2 reviews for further explanation:

Reviewer 1:

In this manuscript, the authors show that the performance of rats trained to discriminate between the intensities of a strong and weak sucrose concentration in an operant task, as well as the resulting intensity generalization function, is unaffected by the conditioning of a taste aversion to sucrose. This is a very important result from a nicely designed experiment because it provides evidence for a dissociation of function between hedonic responsiveness (i.e., the CTA) and sensory-discriminative responsiveness (i.e., the operant intensity discrimination/generalization task). The data are compelling. Its significance with respect to neural processing in the gustatory system and sensory function in general is appreciable.

The main problem with the manuscript is the writing. The text suffers from a lack of precision regarding psychophysical concepts, anthropomorphic assumptions about perceptual experience, and some over-reaching interpretations. Although these shortcomings are serious, they are, fortunately most certainly addressable.

MAJOR

1. Abstract, sentence 1 (and many similar ones throughout the manuscript). “Sucrose’s intensity (its perceived sweetness) and palatability (its positive hedonic valence associated with the act of ingestion) are two taste attributes that increase its attractiveness and promote its overconsumption.

I have a problem with this statement. “Intensity” is generally considered a prothetic characteristic associated with sensory-discriminative function. “Palatability” generally increases or decreases with concentration (or with some artificial sweeteners it can be nonmonotonic), but, in some cases, as nicely shown in this article, changes in palatability are independent of intensity changes. I strongly suggest changing the word “intensity” to concentration and reframing the rest of the sentence in terms of intensity vs. palatability as concentration changes.

2. Throughout the manuscript, the authors claim that they are measuring “perceived sweetness”. This is not precise. For one thing, we don’t know what “sweetness” is to a rat. Moreover, the task was not at all designed to measure anything about quality. The only thing varied was concentration. There is sufficient evidence, both neural and psychophysical, to suggest that the authors were measuring perceived intensity, but there should not be a qualitative label, like “sweetness” associated with the term.

3. Introduction, sentence 1. Related to comment 2, this sentence (and others like it) needs to be rephrased. Rats cannot report sweetness and bitterness. Thus, reword in terms of ligands that are sweet-tasting and bitter tasting to humans.

4. Introduction, sentence 2. This sentence implies that decerebrate rats can identify taste stimuli. At best, the degree to which a decerebrate can identify taste qualities is entirely unknown and, at worst, simply not true. All that is known is that such animals display normal concentration-dependent oromotor reflexes to taste compounds with positive and negative hedonic valence. This is hardly evidence of sensory-discriminative identification. Indeed, making such a claim undermines the principal point of the authors.

5. Page 2, line 5. What is meant by “This consuming experience...”?

6. Page 2, lines 13-17. This kind of logic is flawed and filled with assumption. The basic concept is spot on, but the way it is being conveyed is deeply flawed and characterizes the way much of the text, including the abstract, read. First, as noted above and below, perceived “sweetness” is not being measured. In fact, we don’t know whether the true quality of the stimulus is “sweetness” for the rat to begin with and the intensity discrimination task does not assess quality. Second, although the task used does involve perceived intensity, it is different than simply measuring just noticeable differences (JNDs) in which a simpler claim about perceived intensity could be made. In the latter case, a standard intensity (e.g. a specific concentration) is applied and comparison intensities (e.g., comparison concentrations) are presented and the subject must judge whether the comparison stimuli are different from the standard (see Gescheider GA (1997) Psychophysics: The Fundamentals, 3rd Ed. Mahweh, NJ: Lawrence Erlbaum Associates; and Colbert CL, Garcea M, Spector AC (2004) Effects of selective lingual gustatory deafferentation on suprathreshold taste intensity discrimination of NaCl in rats. Behav Neurosci 118:1409-1417.). In the authors’ task, the rat is trained to respond one way to one standard intensity and another way to a second standard stimulus. Thus, when the rat is presented with test stimuli, it is reporting the similarity of the test stimulus to either the lower or higher intensity. Arguably, responsiveness depends on perceived intensities, but not in a way that will provide clear data on JNDs. I strongly recommend that the entire manuscript be reframed in terms of what is measured. It will make the logic more defensible without taking anything away from the basic and important point of the dissociation in sensory-discriminative vs. hedonic responsiveness.

7. Page 3, lines 2-3. Another way that palatability of a taste stimulus is assessed is through oromotor and somatic taste reactivity and some key citations should be listed here. Moreover, while the citations presented are accurate, those studies measured licking in the context of the microstructure and/or meal patterns of intake tests. While indeed they are appropriate to cite, a very common assay for the palatability of a taste stimulus is the brief access test and that should be mentioned as well. A methodological paper that reviews all of these would perhaps be useful. One possibility is: St. John SJ, Spector AC. (2008) Behavioral analysis of taste function in rodent models. In: The Senses: A Comprehensive Reference, series edited by A. I. Basbaum, A. Kaneko, G. M. Sheppard, and G. Westheimer, 4: Olfaction and Taste, volume edited by S. Firestein and G. K. Beauchamp, San Diego: Academic Press, pp. 409-428.

8. Page 3. Lines 6-8. “However, to have access to the subject sucrose-intensity perceptual representation, we developed a psychophysical task where animals were required to report the sweetness of the sucrose concentration they perceived (Fonseca et al., 2018).” First, once again, the task is not measuring perceived sweetness, it is measuring intensity discrimination; there is nothing about the design that provides information about a quality assessment. See comment #6. Also, to my knowledge, the very first development and application of a suprathreshold taste intensity discrimination procedure in rodents was published by Colbert et al, 2004, and, although the task the authors used has been modified to produce a generalization function, the Colbert et al, 2004 should be given proper attribution.

9. Page 3. Sentence before Materials and Methods. Change “...indicating that the hedonically positive aspects of palatability associated with the oromotor aspects of eating are not necessary for the animals to detect a tastant’s intensity.” To “...indicating that an hedonic evaluation of a taste stimulus is dissociable from its intensive-discriminative properties.”

10. Materials and Methods. It would be helpful to have some more information regarding the behavioral method and analysis. Was there a limited hold (i.e. a time requirement for the animal to respond)? If so, what was it? What was done if the animal did not respond? Were trials without a response counted as errors or not included in analysis? What volume was the fluid drop? Curves were apparently fit to the mean performance. Were curves also fit to each individual’s performance? What was the equation used? Define how the Point of Subjective Equality (PSE) was determined; was it the EC50 of the curve fit? If so, it would be useful to use the EC50s from the individual fits as scores and test whether there was a change from before to after the CTA. How many and which sessions went into the pre and post analysis should be explicitly stated in the methods section.

11. Means and sem are used for virtually all of the figures (Figure 4 and Ex Fig 2.1 are unspecified) , but medians and interquartile ranges are used for Table 1. Also, the analyses of the data sets switches between parametric and nonparametric statistics. The rationale for these analytical and presentation decisions/disparities needs to be articulated.

12. Page 7, lines 19-20. It is absolutely debatable whether Cue Dependent Licks (CDL) are a measure of palatability given that the taste stimulus is serving as a discriminative signal that directs operant responding motivated by water deprivation and reinforced with water. Indeed, despite the prior work cited, there is no evidence that CDLs are a measure palatability in the current data set given that there was no difference between in CDLs between widely disparate sucrose concentrations. Categorical statements such as this detract from the objectivity of the interpretative framework.

13. Extended Data Fig 1-1A (and Fig 2B). There are problems with this figure. First, all the vertical axis origins of all the graphs should be 0; otherwise the visual representation of the magnitude of effects is distorted. Second, all the graphs should be scaled similarly (i.e., have the same vertical axis maximum). Third, there need to be more y-axis tick labels to give the viewer a better idea of what the values actually are.

14. How many trials per session were there across sessions? In particular, did this change after CTA?

15. Figure 2c. The horizontal axis is a log scale, which is appropriate, but that should be mentioned in the caption. Also, because it is on a log scale there should be a break in the axis between 0 and 3%.

16. Page 8, lines 19-20. This sentence is overstated. The word “accurate” is not appropriate because it assumes that the authors can measure the perceptual intensity of sucrose for the rats, which they can’t. What these data show is that responding can be brought under concentration-dependent control when taste is used as a discriminative stimulus.

17. Page 9, bottom. The way that the limen, PSE, and Weber fraction were calculated need to be explicitly defined in the methods. While the PSE can be considered the EC50 of the curve fit, the definition of the Weber Fraction and the limen is unclear to me because of the nature of the task. The task involved discriminating between two concentrations, not discriminating a difference from a single standard concentration. Thus, all these parameters are dependent on which two concentrations are chosen. This does not detract from the lack of effect that CTA had on performance, but it does raise questions about the use of such psychophysical terms.

18. Complete statistical outcomes including the test statistic value and the degrees of freedom should be presented in the text, not just p-values.

19. Page 11, lines 13-16. This is very awkwardly worded. What has been shown is that the animal’s response to the intensity of a stimulus, as measured in this task, is not altered by changing the palatability of the stimulus.

20. Page 11, line 19. What does “multiple representations of sweetness” mean?

21. Page 11, bottom. The evidence that GC is responsible for taste recognition is highly controversial and is entirely inconsistent with a number of studies.

22. Page 12, top. The notion that decerebrate rats process palatability is entirely misguided. The oromotor responses to which the authors refer are basic reflexes. Yes, they can be modulated by forebrain processes, but without the forebrain, those reflexes remain intact. Indeed, a CTA cannot be conditioned in decerebrate rats. Thus, referring to these findings as representing that palatability is processed in the caudal brainstem is not a parsimonious interpretation.

MINOR

23. Abstract, line 7. Change “positively” to “positive”.

24. I suggest that the use of the possessive (i.e., apostrophe’s) be reduced and reworded throughout the manuscript.

25. Page 4, line 17. Change “white noise” to “masking noise”. “White noise” has very specific auditory features to it and unless the authors are certain it conforms to that definition, it is best to be less specific.

26. Page 4, line 18. Change “noise” to “sounds"

27. Page 5, line 3. Change “identifying” to “sampling”. The word “identifying” is an assumption.

28. Page 8, line 22. A better reference would be: St. John SJ, Spector AC. (2008) Behavioral analysis of taste function in rodent models. In: The Senses: A Comprehensive Reference, series edited by A. I. Basbaum, A. Kaneko, G. M. Sheppard, and G. Westheimer, 4: Olfaction and Taste, volume edited by S. Firestein and G. K. Beauchamp, San Diego: Academic Press, pp. 409-428.

29. Page 9, line 2. Change “gastric” to “visceral”.

Reviewer 2:

Summary

Tastes are complex sensory signals, containing information about quality, intensity, and palatability. Under normal circumstances, increasing the intensity of sucrose (i.e. sweetness) correlates with increasing palatability (i.e. pleasantness/hedonic value). In the present study, the authors sought to determine whether the perceptual judgement of the intensity of sucrose is dependent upon the perceptual judgment of its palatability. Using a self-initiated “Yes/no” choice task, the authors trained rats to discriminate between High and Low concentrations of sucrose. After reaching criterion, rats were given a drop of one of six stimuli (five concentrations of sucrose or water) and required to choose whether it was High or Low. Next rats underwent a CTA to High sucrose and were then retested in the discrimination and generalization “Yes/no” choice task. Despite the change in the palatability of sucrose, the rats performance after CTA was unchanged. These findings indicate that the perceptual judgement of sucrose intensity can be dissociated from its hedonically pleasant palatability.

The study’s findings are robust and clear. However, I believe a more complete description of the experimental design and data analyses, as well as further discussion would make for a stronger manuscript. Furthermore, the paper would benefit from a grammar check, as there are numerous instances of typos and/or improper use of grammar.

The findings clearly disentangle perceptual intensity from palatability.

Abstract

1. The authors write, “the subjects’ accuracy to identify sucrose concentrations remained unchanged, even though its palatability was changed from hedonically positive to negative.” Would a more accurate statement be “the subjects’ performance remained unchanged...”, since rats were trained to indicate whether a stimulus was either High or Low and accuracy implies responses indicating specific concentrations of stimuli?

Introduction

The results convincingly show that the rats judgment of sucrose concentrations were similar after the shift in palatability. However, this statement is misleading. “In sum, we found that the subjects perceptual judgments of sucrose concentrations were based solely on the information provided by the intensity attribute...”. Would a more accurate statement be “...that the intensity attribute was sufficient to inform the perceptual judgments of sucrose concentrations”?

Methods

1. Although it is summarized in the ‘Animals’ section, reporting when and how long water was available for each task is easier to follow. The authors did this for the ‘CTA section’ of the methods and it was clear and straightforward. Please clarify.

2. The authors employ a relatively new procedure and more information about the task would be helpful.

a. How long were the discrimination and generalization sessions? Was a session constrained by time or number of trials?

b. Were incorrect choices always paired with 50ms light-off? Or was this only during training?

c. If incorrect choices were always paired with the 50ms light-off (including during the 80% of trials during the generalization task), what was the process during the other 20% of generalization trials. Since none of the generalization trials were rewarded, was the choice paired with a 50ms light out? One could see how the 50ms light-off, accompanied by no reward, could serve as a multisensory cue signaling an incorrect choice. After significant training, the absence of the light-off cue accompanied with no reward could be confusing. Please clarify.

3. This statement is incorrect, “Generalization trials, the subjects received one drop of any of six sucrose concentrations (3, 4.75, 7.5, 11.75, and 18%).”. The task employed 5 sucrose concentrations and water. Please rectify.

4. In the data analysis section, please add text describing the calculation of the point of subjective equality (PSE), the limen, and the Weber fraction.

Results

1. The authors describe the task as being divided into four epochs, then list five (Return, Pre-Cue, CDL, Response, and Outcome). Please clarify.

2. The authors state that the rats “detected the absence of reward in less than 300 ms”. Could the significant difference in licking between rewarded and unrewarded trials, as well as the speed of detection, be influenced by the 50ms light-off cue? Please clarify.

3. For the Day1 and Day 2 24 hour two-bottle tests, was there a difference in the total volume consumed between Control and CTA groups?

Discussion

While I understand that the results of this experiment do not directly examine taste coding in gustatory cortex, the author’s discussion of the electrophysiological and optogenetic experiments are overly narrow.

I appreciate the author’s interpretation of the electrophysiological studies showing that the temporal representation of tastes (first of quality then palatability) by neurons in GC implies a serial processing of information. However, the temporal representation of information does not necessarily equate to serial representation. One does not have to drive the other. One could argue that a dynamic network of parallel circuits could provide information to a cortical region differently over time. For example, the study by Samuelsen et al., 2013 found that inactivation of VPMpc disrupted taste coding in a subpopulation of GC neurons, but did not eliminate it. These findings support a parallel processing mechanism for input to GC.

The work by Wang et al. 2018 relies upon a single experiment (from the same lab) suggesting that anterior insular cortex (aIC) is a “sweet cortical field” and the posterior insular cortex (pIC) is a “bitter cortical field”. The findings of most, if not all, electrophysiological experiments fail to support a topographic separation of taste quality by cortical fields. Furthermore, recent imaging studies (Fletcher et al., 2017, Chen et al., 2020) find that the representation of taste quality is spatially distributed across insular cortex. An equally plausible explanation for the findings by Wang et al. 2018 is that pIC is involved in rejection of negative stimuli regardless of taste quality.

A more thorough discussion of alternative possibilities is necessary if the authors want to discuss their findings in relation to cortical processing of taste.
